# Supplementary material for: Astrocytic chloride is brain state dependent and modulates inhibitory neurotransmission in mice
Source: Nat Commun. 2023 Apr 4;14:1871. doi: 10.1038/s41467-023-37433-9 (PMC10073105; doi:10.1038/s41467-023-37433-9)
Supplement: Supplementary file 1 — Supplementary Information [file 41467_2023_37433_MOESM1_ESM.pdf]

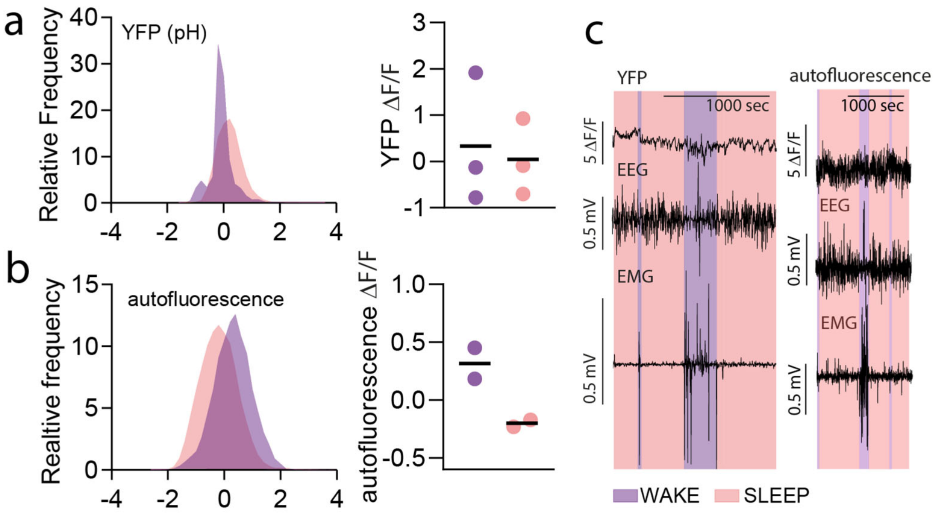

Supplemental Figure 1: In contrast to mClY, YFP or autofluorescence is not affected by brain state changes in vivo.

(a) Distribution of YFP recorded from freely moving intermittently naturally sleeping mice. N = 3 mice. YFP expressed under the *Gfap*-promotor, virally delivered into somatosensory cortex by injection. (b) Distribution of autofluorescence recorded from freely moving intermittently naturally sleeping mice, which do not express any sensor. N = 2 mice. (c) Representative traces of YFP or autofluorescence, EEG, and EMG; colour code highlights brain states sleep and awake. All fluorescent traces were inverted (-ΔF/F%). Data represent mean. Source data are provided as a Source Data file.

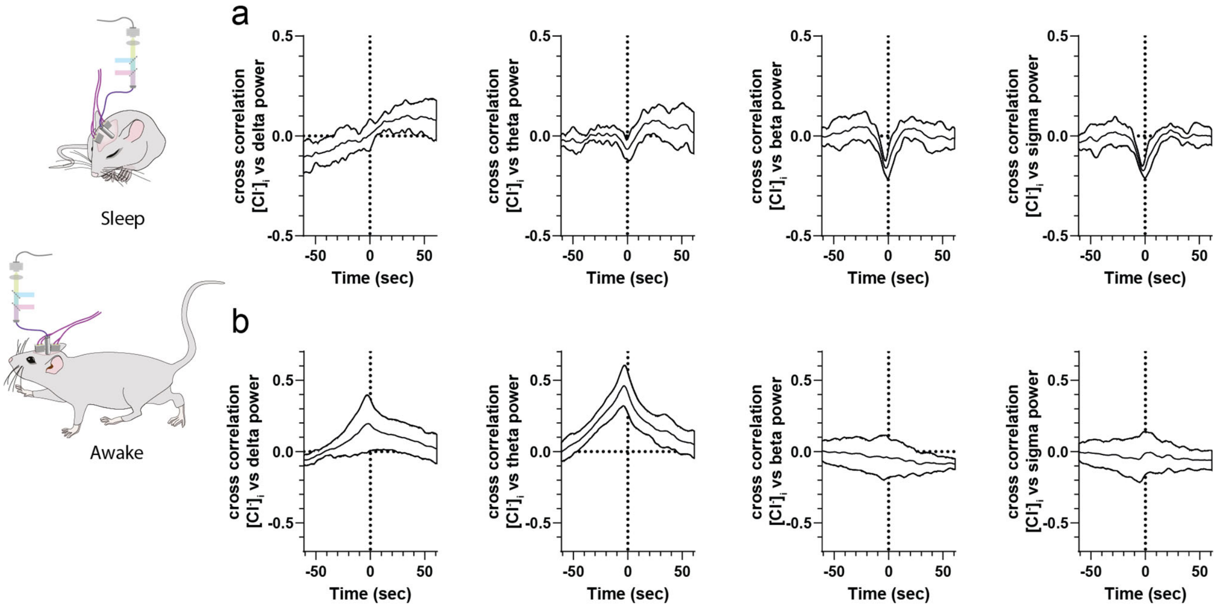

Supplemental Figure 2: Cross correlation of astrocytic $[Cl^-]_i$ *and EEG power bands show a correlation between theta power and astrocytic* $[Cl^-]_i$ *during wakefulness with no correlation during sleep.*

Cross correlation of astrocytic $[Cl^-]_i$ during sleep (a) or wakefulness (b) and EEG power bands, delta 0.2-4 Hz, theta 4-7 Hz, beta 8-15 Hz, sigma 15-30 Hz. N = 5 mice, error±SD.

The average Pearson correlation coefficient: (a) delta: r=0.01, theta r= 0.1, beta r=0.16, sigma r=0.17 (b) delta r=0.18, theta r=0.45, beta r= 0.06, sigma r=0.01. Source data are provided as a Source Data file.

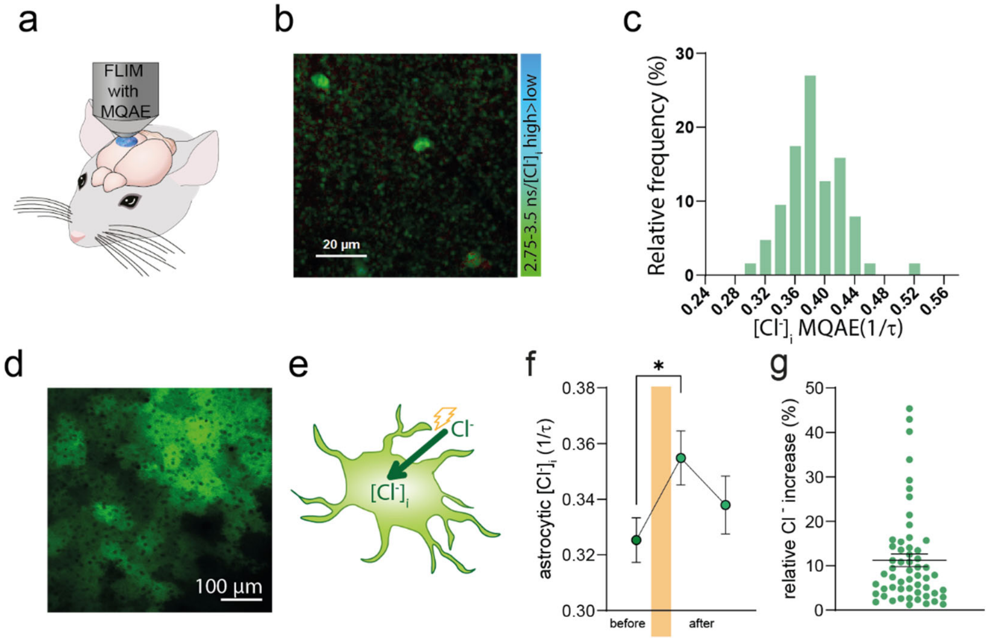

Supplemental Figure 3: Absolute $[Cl^-]_i$ using MQAE and fluorescence lifetime microscopy imaging (FLIM) in combination with optogenetic manipulation of astrocytic $[Cl^-]_i$ to significantly increase astrocytic $[Cl^-]_i$.

(a) Experimental protocol: 2PM fluorescence lifetime imaging (FLIM) of astrocytic $[Cl^-]_i$ using the fluorescent dye MQAE in awake head-fixed mice. (b) Representative FLIM imaged of MQAE loaded cortical astrocytes. Colour code indicates fluorescence lifetime. (c) Distribution of fluorescence lifetime (τ) determined in astrocytes representing absolute $[Cl^-]_i$ shows a normal distribution of $[Cl^-]_i$. N = 6 mice. (d) Representative 2PM intensity imaged of NpHR3.0 expressed by cortical astrocytes. (e) The optogenetic tool NpHR3.0 is a light activated Cl^- pump, actively pumping Cl^- into astrocytes. (f) Average astrocytic $[Cl^-]_i$ upon optogenetic stimulation of NpHR3.0 is significantly increased. N = 3 mice. After is the accumulated signal of 0-21 seconds and 22-42 seconds after turning off the light source. Un-paired two-tailed t-test, *P=0.0238 (g) Relative change of astrocytic $[Cl^-]_i$ upon optogenetic stimulation. N = 5 mice, 56 cells. Data represent mean±SEM. Source data are provided as a Source Data file.

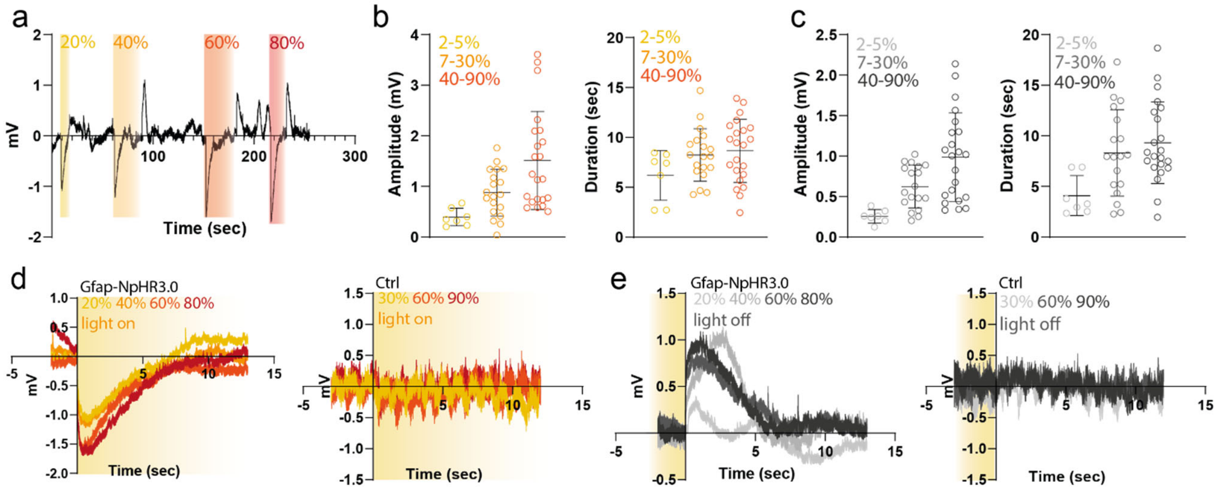

Supplemental Figure 4: Optogenetic stimulation of astrocytes using NpHR3.0 changes $[Cl^-]_i$ in a stimulation light intensity dependent manner.

(a) Representative local field potential (LFP) of brain activity upon light stimulation with different power 20, 40, 60, and 80%. (b and c) Comparison of amplitude and duration of LFP signal upon activation and deactivation of light stimulation, indicate a light intensity dependent NpHR3.0 transport current. N = 1 mouse, 7 recordings 2-5%, 4 mice, 19 recordings 7-30%, 3 mice, 22 recordings 40-90%. (d and e) Representative traces of NpHR3.0 transport current upon activation and deactivation of light stimulation, and representative traces of negative control not expressing NpHR3.0 upon activation and deactivation of light stimulation. N = 4 mice. Data represent mean±SD. Source data are provided as a Source Data file.

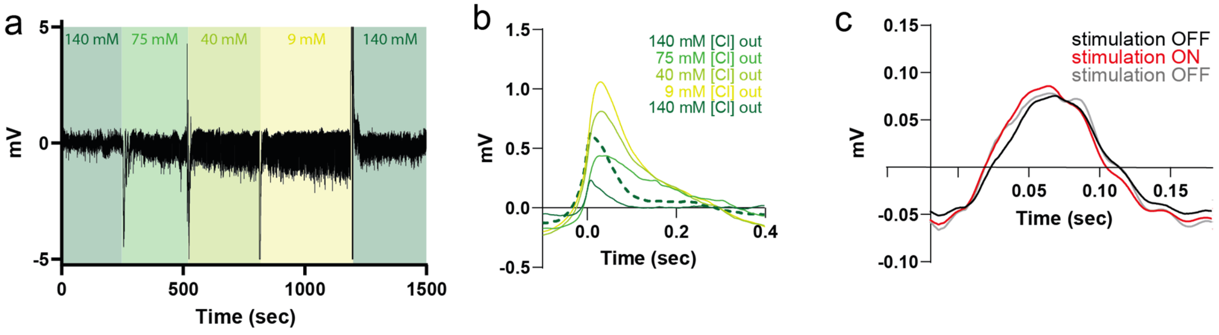

Supplemental Figure 5: In contrast to optogenetic manipulation of astrocytic $[Cl^-]_i$, manipulations with $[Cl^-]_o$ do affect spontaneous neuronal activity.

(a) Representative LFP recording from the cortex with aCSF containing different [Cl^-] (0, 40, 75, or 140 mM), showing the impact of changed [Cl^-]$_o$ on spontaneous neuronal activity. (b) Waveform average of LFP recorded with aCSF containing different [Cl^-]. (C) Waveform average of LFP with and without activation of NpHR3.0 in astrocytes shows no change of spontaneous neuronal activity upon optogenetic stimulation. N = 2 mice, 6 recordings. Data represent mean±SD. Source data are provided as a Source Data file.

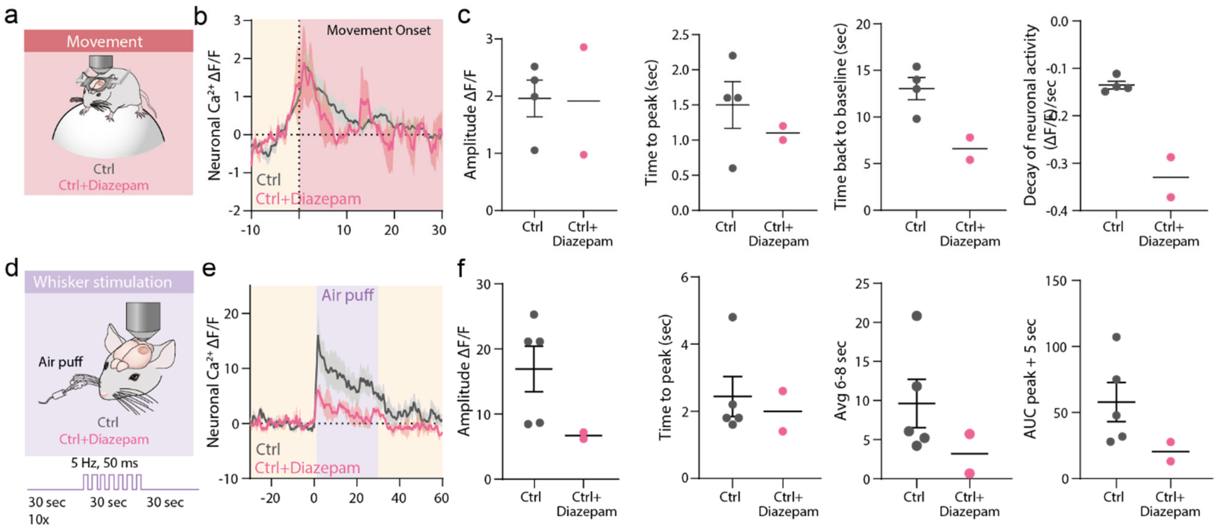

Supplemental Figure 6: GABA$_A$R activator Diazepam, which increases GABAergic inhibition, supresses the neuronal activation upon movement onset and whisker stimulation.

(a) Experimental protocol: Neuronal [Ca^{2+}]$_i$ was imaged using jRGECO expressed under the neuronal *hsyn* promoter, while pharmacologically stimulating GABA$_A$R using Diazepam. The awake, head-fixed mice were voluntarily running on a Styrofoam sphere. (b) Average neuronal [Ca^{2+}]$_i$ trace during transition from stationary to mobile, in absence and in presence of Diazepam, shading indicates±SEM. N = 4 ctrl/ 2 Diazepam. (c) Maximal (peak) amplitude of neuronal [Ca^{2+}]$_i$ transient upon movement onset. Time to peak of neuronal [Ca^{2+}]$_i$ transient upon movement onset. Recovery time of neuronal [Ca^{2+}]$_i$ transient back to baseline. N = 4 mice ctrl, N = 2 mice Diazepam. Decay constant of neuronal [Ca^{2+}]$_i$ transient upon movement onset. (d) Using the same

pharmacological activation of GABA$_A$R and neuronal $[Ca^{2+}]_i$ imaging as in (a), whiskers were stimulated using air puffs. (e) Average neuronal $[Ca^{2+}]_i$ trace during whisker stimulation, while simultaneously activating the optogenetic tool NpHR3.0 in astrocytes, shading indicates±SEM. N = 5 mice ctrl/ 2 mice Diazepam. (f) Maximal (peak) amplitude of neuronal $[Ca^{2+}]_i$ upon whisker stimulation; time to peak of neuronal $[Ca^{2+}]_i$ upon whisker stimulation. Neuronal $[Ca^{2+}]_i$ during the period of maximal astrocytic $[Cl^-]_i$ changes upon whisker stimulation, 6 – 8 sec after onset of stimulation. AUC of neuronal $[Ca^{2+}]_i$ during 5 sec after peak. Data represent mean±SEM. N = 5 mice ctrl/ 2 mice Diazepam. Source data are provided as a Source Data file.

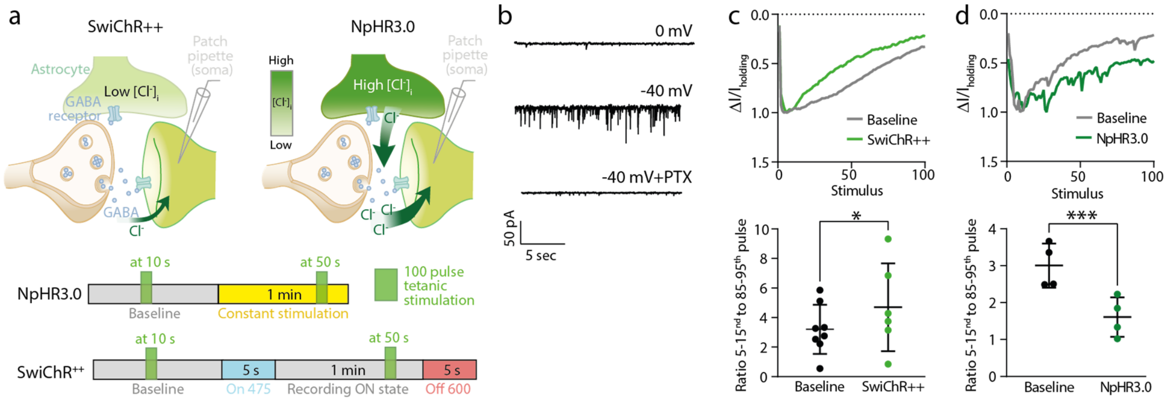

Supplemental Figure 7: Optogenetic manipulation of baseline astrocytic $[Cl^-]_i$ affects activity-dependent depolarizing shifts of IPSC.

(a) IPSC was recorded in acute cortical slices upon electrical stimulation as described before[38]. (b) To study GABA$_A$ receptor antagonist-insensitive currents, recordings were made in the presence of 200 µM picrotoxin (PTX) or at 0 mV holding potential. (c) In controls, the IPSC of the 90th stimulus (average of 85-95th stimulus) is smaller compared to the 10th stimulus (average of 5-15th stimulus) which reflects the collapse of the Cl gradient. In contrast, activation of NpHR3.0 in astrocytes causing an increase in baseline astrocytic $[Cl^-]_i$ results in a slower run-down of the IPSCs. The higher 90th stimulus indicates a longer maintenance of the Cl^- gradient. Paired two-tailed t-test, *P=0.0357 (d) The optogenetic tool SwiChR++, which is switchable Cl^- channel

decreases baseline $[Cl^-]_i$ in astrocytes. Activation of SwiChR++ increases the ratio between 10th and 90th response indicative of a faster rundown of the response to stimulation. (N = 4 mice, 4 recordings NpHR3.0, 5 mice SwiChR++, 8 recordings baseline, 6 recordings SwiChR++) Paired two-tailed t-test, **P=0.0004. All recordings were performed in presence of CNQX (20 µM), D-AP5 (50 µM) and CGP55845 (3 µM), tetanus stimulation (100 times at 100 pA, 200 µs, 50 Hz). Data represent mean±SEM. Source data are provided as a Source Data file.

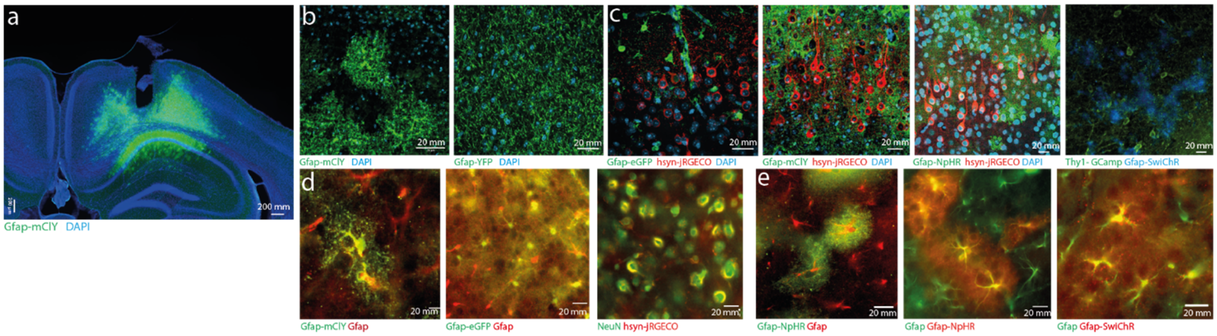

Supplemental Figure 8: Validation of cell specificity of biosensor expression.

(a) Position of the tip of the fibre optics used for fibre photometry. (b) Expression of Gfap-mClY and Gfap-YFP in cortical astrocytes (green). (c) Expression of Gfap-eGFP, Gfap-mClY or Gfap-NpHR3.0 (green) together with hsyn-jRGECO (red). Nuclei are labeled with DAPI (blue). Expression of Thy1-Gcamp (green) and Gfap-SwiChR++ (blue) (d and e) Colocaliszation of cell specific marker (astrocytes = Gfap or neurones = NeuN) and injected biosensor or optogenetic tool. All stainings have been repeated in three individual animals.

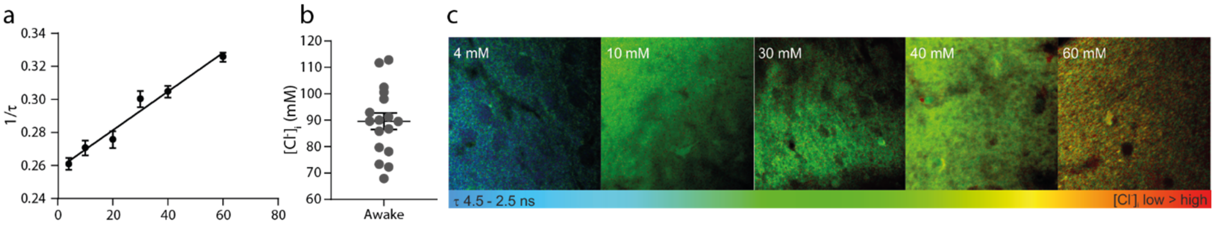

Supplemental Figure 9: Calibration of MQAE fluorescence lifetime in brain slices.

(a) Stern Vomer plot of the calibration of Cl^- dependence of MQAE fluorescence lifetimes in acute brain slices. Data represent mean±SEM. N = 4 mice. (b) Translation of *in vivo* recorded fluorescence lifetimes into absolute Cl^- concentration based on the *in situ* calibration curve. Data represent mean±SEM. N = 4 mice, 38 cells). (c) Color coded fluorescence lifetime images of the different Cl^- concentration calibrated. Limitation of the calibration: Cl^- sensitivity of MQAE is pH independent and not affected by other ions, however dye hydrolysis, self-quenching and non-specific quenching by cytosolic proteins are cell type and sample specific. Therefore, the calibration performed in acute brain slices cannot reliably translate recordings obtained *in vivo*[1-3]. Source data are provided as a Source Data file.

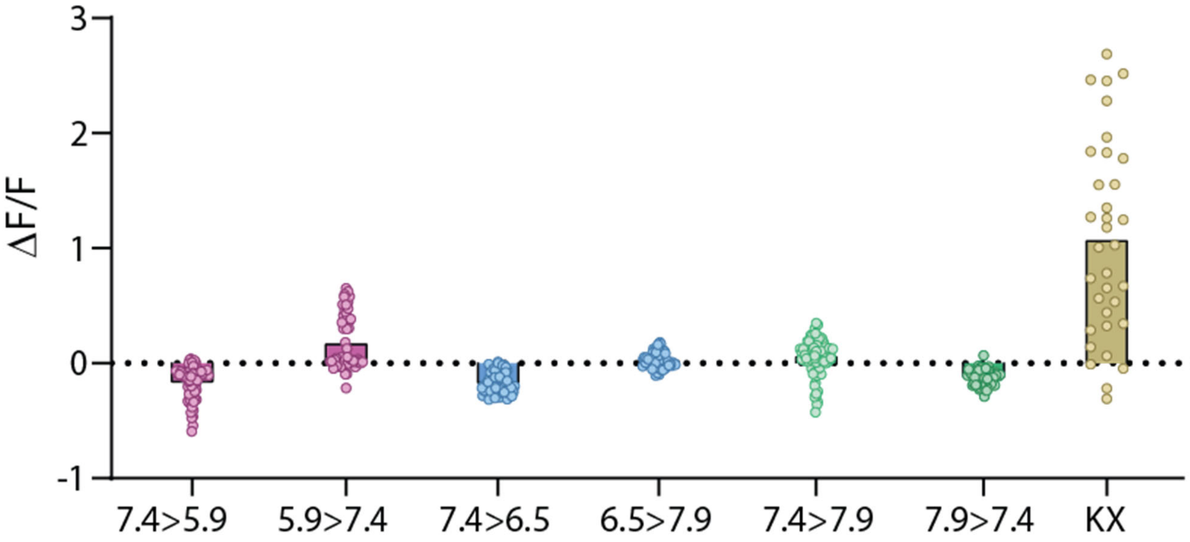

Supplemental Figure 10: mClY expressed in vivo astrocytes is pH insensitive.

Fluorescence intensity of mClY expressed in astrocytes was imaged via an acute craniotomy using 2-photon microscopy. aCSF containing different pH was perfused while the change of fluorescence intensity of mClY was imaged. The values on the x-axis indicate the pH changes (pH before> pH after) KX indicates the change from awake to KX anaesthesia. As a control the animal was anesthetised using ketamine xylazine anesthesia. N = 1 animal, 71 cells 7.4>5.9/ 59 cells 5.9>7.4/

70 cells 7.4>6<5/ 73 cells 6.5>7.9/ 80 cells 7.4>7.9/ 64 cells 7.9>7.4/ 34 cells KX. Data represent mean±SEM. Source data are provided as a Source Data file.

Supplementary Table 1: Astrocytes express multiple subunits of $GABA_A$ receptors[4,5] (https://portal.brain-map.org/).

Allan 10x single cell - whole cortex and hippocampus					Heintz data base summary Doyle et al 2008 Cell Ribotrap Aldh1L1 Astrocytes Cortex Normalized			Ben Barres Brain RNA-Seq Li et al 2018		
Gene	protein nam	x378_Astro	x376_Astro	x377_Astro		variant	protein name	Mus musculus	Homo sampiens -	Homo sampiens - fetal
**'Gabra1'**	alpha1			1.2700	465.9412674	BQ268470		0.1±0	0.1±0	0.1±0
					415.8488542	BE945884				
					10.05093654	Z36357				
					5.298788811	Z36357				
**'Gabra2'**	alpha2	1.9274	3.5516		**390.3840247**	BB339336		**15.2393±3.4944**	46.0606±3.6761	0.8997±0.3332
					**120.0944862**	BQ174589				
					**76.47509475**	NM_008066				
					8.462597899	AV379247	AV379247 RIKEN full-length enriched, adult			
					6.728615808	AV379247	AV379247 RIKEN full-length enriched, adult			
					5.083145333	BB433285	Transcribed sequences			
**'Gabra3'**	alpha3				4.906019269	NM_008067		0.1666±0.0536	0.6547±0.0968	2.5734±1.3504
**'Gabra4'**	alpha4				**1628.359824**	BB430205		**12.4015±0.8353**	2.6121±0.5117	0.2129±0.0574
					**182.3475983**	AK013727				
**'Gabra5'**	alpha5				32.56755474	BQ175863				
**'Gabra6'**	alpha6				6.324737669	NM_008068		0.1±0	0.1±0	0.1±0
					5.617565322	AF256198				
**'Gabrb1'**	beta1	9.9478	11.2202	3.4285	**3051.028922**	NM_008069		**18.062±3.8636**	28.9188±1.9468	3.3455±1.7409
**'Gabrb2'**	beta2				8.160633955	NM_008070		**15.2393±3.4944**	46.0606±3.6761	0.8997±0.3332
**'Gabrb3'**	beta3				159.8195906	BQ175666		0.9581±0.1662	3.4870±0.2149	2.5916±0.4320
					12.76251916	BB367779				
					5.514779964	BB367779				
**'Gabrd'**	delta				6.461331392	BE946994		0.1±0	0.1±0	0.1±0
					5.260348726	NM_008072				
**'Gabre'**	epsilon				5.358142582	NM_017369		0.1614±0.0614		
**'Gabrg1'**	gamma1	0.3147	0.6147		**4517.096667**	AF156490		37.6960±4.3232	16.882±1.5497	0.6184±0.3926
					**322.9320861**	AF156490				
					4.765074503	BE946928	Transcribed sequence with weak similarity			
**'Gabrg2'**	gamma2				15.26860289	AV348821		0.1424±0.0234	0.1905±0.0445	0.0987±0.0012
					4.684561598	AF233778				
**'Gabrg3'**	gamma3				5.860644796	NM_008074		2.3917±0.1268	0.1003±0.000325	0.1±0
**'Gabrp'**	pi				6.089978571	BC027245		0.1±0	0.1±0	0.1098±0.0098
					4.116624965	BC027245				
**'Gabrq'**	theta				5.074559857	NM_020488		0.1±0	0.2520±0.0712	0.4275±0.2106
**'Gabrr1'**	rho1				5.258867444	NM_008075		0.1±0	0.1±0	0.1±0
**'Gabrr2'**	rho2				5.317823179	AF024621		0.2917±0.0375	0.1044±0.0044	0.1±0
**'Gabrr3'**	rho3							0.1±0	0.1017±0.0017	0.1±0
**'Aqp4'**		10.9980	3.9287	3.6354	15696.61217	BB193413	Adult male spinal cord cDNA, RIKEN full-len	317.6387±15.0845	449.9831±36.4463	14.0190±6.3434
					823.2821282	U48399				
					113.8031517	AW489155				

References

1 Gensch, T., Untiet, V., Franzen, A., Kovermann, P. & Fahlke, C. in *Advanced Time-Correlated Single Photon Counting Applications* Vol. 111 *Springer Series in Chemical Physics* (ed Wolfgang Becker) Ch. 4, 189-211 (Springer International Publishing, 2015).

2 Kaneko, H., Putzier, V., Frings, S. & Gensch, T. in *Calcium-Activated Chloride Channels* Vol. 53 *Current Topics in Membranes* (ed C. M. Fuller) 167-+ (Elsevier Academic Press Inc, 2002).

3 Verkman, A. S., Sellers, M. C., Chao, A. C., Leung, T. & Ketcham, R. Synthesis and characterization of improved chloride-sensitive fluorescent indicators for biological applications. *Analytical biochemistry* **178**, 355-361 (1989).

4 Doyle, J. P. *et al.* Application of a translational profiling approach for the comparative analysis of CNS cell types. *Cell* **135**, 749-762 (2008). https://doi.org:10.1016/j.cell.2008.10.029

5 Li, Q. *et al.* Developmental Heterogeneity of Microglia and Brain Myeloid Cells Revealed by Deep Single-Cell RNA Sequencing. *Neuron* **101**, 207-223 e210 (2019). https://doi.org:10.1016/j.neuron.2018.12.006
