# Supplementary material for: Astrocytic chloride is brain state dependent and modulates inhibitory neurotransmission in mice
Source: Nat Commun. 2023 Apr 4;14:1871. doi: 10.1038/s41467-023-37433-9 (PMC10073105; doi:10.1038/s41467-023-37433-9)
Supplement: Supplementary file 3 — Reporting Summary [file 41467_2023_37433_MOESM3_ESM.pdf]

nature portfolio

Corresponding author(s): Maiken Nedergaard, Alexei Verkhratsky, Verena Untiet

Last updated by author(s): Feb 17, 2023

Reporting Summary

Nature Portfolio wishes to improve the reproducibility of the work that we publish. This form provides structure for consistency and transparency in reporting. For further information on Nature Portfolio policies, see our Editorial Policies and the Editorial Policy Checklist.

Statistics

For all statistical analyses, confirm that the following items are present in the figure legend, table legend, main text, or Methods section.

n/a	Confirmed	
☐	☒	The exact sample size (*n*) for each experimental group/condition, given as a discrete number and unit of measurement
☐	☒	A statement on whether measurements were taken from distinct samples or whether the same sample was measured repeatedly
☐	☒	The statistical test(s) used AND whether they are one- or two-sided<br>*Only common tests should be described solely by name; describe more complex techniques in the Methods section.*
☒	☐	A description of all covariates tested
☐	☒	A description of any assumptions or corrections, such as tests of normality and adjustment for multiple comparisons
☐	☒	A full description of the statistical parameters including central tendency (e.g. means) or other basic estimates (e.g. regression coefficient) AND variation (e.g. standard deviation) or associated estimates of uncertainty (e.g. confidence intervals)
☐	☒	For null hypothesis testing, the test statistic (e.g. *F*, *t*, *r*) with confidence intervals, effect sizes, degrees of freedom and *P* value noted<br>*Give P values as exact values whenever suitable.*
☒	☐	For Bayesian analysis, information on the choice of priors and Markov chain Monte Carlo settings
☒	☐	For hierarchical and complex designs, identification of the appropriate level for tests and full reporting of outcomes
☐	☒	Estimates of effect sizes (e.g. Cohen's *d*, Pearson's *r*), indicating how they were calculated

Our web collection on statistics for biologists contains articles on many of the points above.

Software and code

Policy information about availability of computer code

Data collection	All software for data collection is detailed in he manuscript. These include: Synapse v. 96 (Tucker Davis Technologies), SleepScore (4.0.0.40 ViewPoint Behavior Technology), Ethovision XT 11.5 (Noldus), NIS-Elements (Nikon), Sciscan (Scientifica), Symphotime(Picoquant), µManager, Thorimage(Thorlabs), pCLAMP 10.2 software (Molecular Devices).
Data analysis	All software for data analysis is detailed in the manuscript. These include: DeepLabCut, Fijii, MatLab, Prism (GraphPad), Symphotime (Picoquant), SleepScore (ViewPoint Behavior Technology), Ethovision XT 11.5 (Noldus).

For manuscripts utilizing custom algorithms or software that are central to the research but not yet described in published literature, software must be made available to editors and reviewers. We strongly encourage code deposition in a community repository (e.g. GitHub). See the Nature Portfolio guidelines for submitting code & software for further information.

Data

Policy information about availability of data

All manuscripts must include a data availability statement. This statement should provide the following information, where applicable:

- Accession codes, unique identifiers, or web links for publicly available datasets
- A description of any restrictions on data availability
- For clinical datasets or third party data, please ensure that the statement adheres to our policy

All relevant source data are provided with the manuscript. Example raw signals are included within the manuscript. Unprocessed electrophysiological recording data

are available from the corresponding authors upon reasonable request.

Human research participants

Policy information about studies involving human research participants and Sex and Gender in Research.

Reporting on sex and gender	N/A
Population characteristics	N/A
Recruitment	N/A
Ethics oversight	N/A

Note that full information on the approval of the study protocol must also be provided in the manuscript.

Field-specific reporting

Please select the one below that is the best fit for your research. If you are not sure, read the appropriate sections before making your selection.

[x] Life sciences [ ] Behavioural & social sciences [ ] Ecological, evolutionary & environmental sciences

For a reference copy of the document with all sections, see nature.com/documents/nr-reporting-summary-flat.pdf

Life sciences study design

All studies must disclose on these points even when the disclosure is negative.

Sample size	Sample sizes were based on accepted stanards in the field. These are sufficient to generate meaningful conclusions given biologically relevant effect sizes and typical data variance for the meaures used. Sample sizes used were compareble to previous studies that used similar techniques (Alfonsa et al 2022 Nat Neurosci doi.org:10.1038/s41593-022-01214-2, Weilinger et al 2022 Cell Rep doi.org:10.1016/ j.celrep.2022.111556, Alfonsa et al 2015 J Neurosci doi.org:10.1523/JNEUROSCI.4105-14.20159).
Data exclusions	No data were excluded.
Replication	Chloride imaging in astrocytes and optogentic manipulation of astrocytic [Cl-]i and its impact on neuronal signaling have been tested using different imaging approaches as well as electrophysiological recordings.<br>1. Brain state dependent astrocytic Cl was recorded in two independent groups, recorded by two different scientists and scored by two different scientists. No data was excluded and replication was successful.<br>2. Astrocytic Cl changes upon movement onset and sensory stimulation was recorded in two independent groups. No data was excluded, replication was successful.<br>3. The effect of optogenetic stimulation of NpHR was recorded in two different groups for Cl imaging as well as neuronal Ca imaging. No data was excluded, replication was successful.<br>4. The effect of optogenetic stimulation of SwiChR was recorded in two different groups for Cl imaging as well as neuronal Ca imaging. No data was excluded, replication was successful.<br>5. The effect of activation of GABAAR on astrocytic Cl was repeated using two different biosenors/fluorescent dyes (mClY and MQAE) as well as two different pharmacological agonists (GABA and muscimol). No data was excluded. replication was successful.
Randomization	Within-animal comparisons were performed whenever possible. Animals in test and control groups were litter mates. Mice were housed in groups of 5, which were randomly selected to become test or controle animal, while each group contained both. Conditions were counterbalanced and randomized across mice.
Blinding	Investigators were blinded to the conditions as far as possible, macroscopic imaging and fibre photometry recordings were blinded. Some experiments were not blinded, because the experimental conditions were obvious to the researchers, this includes 2PM imaging upon optogenetic stimulation. Expression of optogenetic tools was confirmed before imaging and the optimal imaging plane was choosen based on expression of the optogenetic tools. Puff injection of GABA/Muscimol/aCSF was not blinded.<br>All measurements were subsequently analyzed applying the same automated criteria.

Reporting for specific materials, systems and methods

We require information from authors about some types of materials, experimental systems and methods used in many studies. Here, indicate whether each material, system or method listed is relevant to your study. If you are not sure if a list item applies to your research, read the appropriate section before selecting a response.

Materials & experimental systems

n/a	Involved in the study
☐	☒ Antibodies
☒	☐ Eukaryotic cell lines
☒	☐ Palaeontology and archaeology
☐	☒ Animals and other organisms
☒	☐ Clinical data
☒	☐ Dual use research of concern

Methods

n/a	Involved in the study
☒	☐ ChIP-seq
☒	☐ Flow cytometry
☒	☐ MRI-based neuroimaging

Antibodies

Antibodies used

Anti-RFP antibody, 1:500 Abcam AB62341, Mouse anti-neuronal nuclei (NeuN) monoclonal antibody, clone A60, 1:500 Merck Millipore MAB377, Anti-Gfap antibody, 1:500 Thermo Fisher Scientific PA1-10004, Anti-GFP antibody, 1:500 Thermo Fisher Scientific A-6455, Anti-mCherry antibody, 1:500 Thermo Fisher Scientific AB_2536611, Goat-anti-chicken IgY (H+L) Thermo Fisher Scientific A21449 (1:500), Goat-anti-rabbit IgG (H+L) Life Technologies/invitrogen A11034 (1:500), Goat-anti-rabbit IgG (H+L) Thermo Fisher Scientific A11011 (1:500), Goat-anti-mouse-IgG2b Thermo Fisher Scientific A32728 (1:500), Goat-anti-rat IgG (H+L) Abcam AB1755710 (1:500).

Validation

Anti-RFP antibody (AB62341): Used in 237 references, Use at an assay dependent concentration. PubMed: 23300543 for PFA fixed sections. Mouse anti-neuronal nuclei (NeuN) monoclonal antibody (MAB377): Has been published and validated for PFA fixed sections (Sarnat et al. (1998) 10.1016/s0387-7604(97)00111-3, Wang Y, et al. (2022) 10.1016/j.brainresbull.2022.02.019). Anti-Gfap antibody (PA1-10004): Used in 25 references, this Antibody was verified by Relative expression to ensure that the antibody binds to the antigen stated, suitable for PFA fixed sections (Klein A, et al. (2022) 10.1371/journal.pntd.0009845). Anti-GFP antibody (A-6455): Used in 1427 references, applicatoin validated for IHC (Bono BS, et al. (2022) 10.1002/cne.25306). Anti-mCherry antibody (M11217): Used in 129 references (Robert V, et al. (2021) 10.7554/eLife.63352).

Animals and other research organisms

Policy information about studies involving animals; ARRIVE guidelines recommended for reporting animal research, and Sex and Gender in Research

Laboratory animals

Group-housed male and female C57BL/6J mice (from Janvier) and Thy1-GCaMP mice bred in house (heterozygous versus wildtype breeding, purchased from Jackson laboratories, strain 024275) were used. Mice were maintained on a 12:12 light cycle (lights on at 07:00) at 21 degrees C with 40-60% humidity with water and food ad libitum. Mice were 10-20 weeks at time of experiments.

Wild animals

The study did not involve wild animals

Reporting on sex

Sex differences were not considered in this study, animals of both sex were included.

Field-collected samples

The study did not involve samples collected from the fields

Ethics oversight

All experiments conducted at University of Copenhagen were approved by the Danish Animal Experiments Inspectorate and were overseen by the University of Copenhagen Institutional Animal Care and Use Committee (IACUC), in compliance with the European Communities Council Directive of 22 September 2010 (2010/63/EU) legislation governing the protection of animals used for scientific purposes. All experiments conducted at the University of Rochester were approved by the University Committee on Animal Resources of the University of Rochester Medical Center and an effort was made to minimize the number of animals used.

Note that full information on the approval of the study protocol must also be provided in the manuscript.
